# The temporal impact of aging on the burden of herpes zoster

**DOI:** 10.1186/s12877-017-0420-9

**Published:** 2017-01-23

**Authors:** Lijoy Varghese, Baudouin Standaert, Antonio Olivieri, Desmond Curran

**Affiliations:** 1 GSK R&D - Asia-Pacific & North Asia, 150 Beach Road, #22-00 Gateway West, Singapore, 189720 Singapore; 2 GSK, Wavre, Belgium; 3 Current affiliation: Alexion Pharmaceuticals, Lausanne, Switzerland

**Keywords:** Herpes Zoster, Incidence, Temporal, Australia, Japan, United States

## Abstract

**Background:**

The risk of Herpes Zoster (HZ) increases with age and various studies have also demonstrated an increasing HZ incidence globally. Simultaneously, the global trend of an aging population has placed a greater burden on the healthcare system. This study aims to estimate the potential burden of HZ over time accounting for the increasing trends of both HZ incidence and global aging.

**Methods:**

A recent systematic review on HZ incidence identified studies that evaluated the temporal effects of HZ incidence. Data from the identified studies were extracted to estimate the trend of HZ incidence in the ≥65-year-old age cohort. The incidence rates were estimated up to the year 2030 using two scenarios: a linear extrapolation and a last observation carried forward. Three countries were chosen to perform the analysis on: Australia, Japan and the United States.

**Results:**

The incidence data from the three countries showed an average annual increase between 2.35 and 3.74% over the time period of the studies selected. The elderly population for the US, Japan and Australia are expected to increase by 55, 10 and 53% respectively by the year 2030 compared to the levels in 2015. Under the first scenario between 2001 and 2030, the number of annual incident cases of HZ in those aged ≥65 years is expected to increase by +343% (293,785 to 1,303,328), +176% (158,616 to 437,867) and +376% (18,105 to 86,268) in the US, Japan and Australia respectively while those for the second scenario are +150%, +83% and +223% respectively. In the US alone, the estimated annual cost of HZ-related cases in the ≥65 age cohort is approximately 4.74 Billion US$ in 2030.

**Conclusions:**

The increasing incidence of HZ coupled with the demographic trends (i.e., aging population and greater life expectancy) in many countries are likely to imply a rising economic burden of HZ on already constrained healthcare budgets.

## Background

Herpes Zoster (HZ) which is a reactivation of the latent Varicella Zoster Virus (VZV) [1] is primarily a disease in older adults and those with immunosuppressed conditions or treatments [2]. A population based study conducted by Yawn et al. using data between 1996 and 2005 in Olmsted County in the United States (US) reported that the risk of HZ increases with age; from 4.7 per 1,000 in 50–59 year-old to 12.0 per 1,000 in ≥80 year-old [3]. The study also reported that HZ complications, primarily Post-herpetic Neuralgia (PHN, defined as pain lasting more than 90 days from the onset of the HZ rash), also increase with age; from 5.41% in 50–59 year-old to 20.32% in ≥80 year-old [3]. An extrapolation of the HZ incidence rate obtained from the study to the 2005 US population, results in approximately 1 million new cases of HZ each year [3].

Various studies [4–11] have shown that the HZ incidence increased in many countries, e.g. Australia, Canada, Japan and the US. Hales et al. [11] estimated a 39% increase in HZ incidence in the ≥65 year-old US Medicare insured population between 1991 and 2010. Many reasons have been cited for the increasing trend: (1) introduction of routine varicella vaccination that reduces the exposure to varicella and, thus, exogenous boosting [12, 13], (2) increasing aging population and the associated prevalent chronic diseases, (3) increasing prevalence of immunosuppression in the elderly population, and (4) immune suppression brought on by long-term stress and depression [14].

Simultaneously, the global trend of aging populations presents a challenge to healthcare resources especially with respect to diseases that impact the elderly such as HZ. The National Institute on Aging estimates that by 2030, the world is likely to have over 1 billion people aged ≥65. The oldest-old, i.e. the population aged ≥85, is projected to grow by 46% between 2015 and 2030 compared to of 28% for those aged ≥65 [15]. The increasing HZ incidence coupled with the demographic trends (i.e., aging population and greater life expectancy) in many countries would imply a greater economic burden of HZ on already constrained healthcare budgets.

The objective of this study is to estimate the potential burden of HZ (in terms of incident HZ cases) over time by taking into consideration the trends of HZ incidence and global aging. In this paper we focus on three countries for which we project the incident cases of HZ up to 2030 in the ≥65 age cohort; the US, Japan and Australia.

## Methods

A recent systematic review by Kawai et al. on the global HZ incidence identified 25 studies from 7 countries up to December 2013 that presented the temporal trends of HZ incidence [16]. We performed a search on PubMed to identify more recent studies using a similar search strategy [16]. We used Medical Subject Headings (MeSH) terms for ‘herpes zoster’ in combination with the terms ‘incidence’ and ‘epidemiology’ from January 2014 to November 2016. Studies that evaluated incidence of HZ in children or adolescents and those that assessed the rates of hospitalization arising from HZ were excluded. Five new studies that reported temporal trends were identified [17–21].

Data from the included studies were extracted to estimate the annual HZ incidence (per 1,000 persons) in the ≥65 age cohort. If yearly incidence data was reported for different age cohorts, an aggregate age-adjusted incidence value for the ≥65 age cohort was estimated using individual country demographic data obtained from the respective national statistics databases. If the incidence data was further reported based on gender, the age-specific proportion of males to females obtained from the corresponding national population databases was used to arrive at an aggregate value.

To evaluate the future burden of HZ the incidence rates for the various countries were projected up to the year 2030 using the following two scenarios:

- Scenario 1 assumes that the linear trend modeled for the corresponding country-specific annual HZ incidence data is followed till the year 2030. A linear curve was found to be the best-fit for the reported yearly incidence reported in a majority of the data-sets and consequently used for the studies considered.
- Scenario 2 assumes a last observation carried forward value, whereby the predicted incidence based on the best fit linear curve of the last reported year of each study is carried forward (i.e. assuming a constant incidence value from the year of the conclusion of the study till the year 2030). This conservative scenario assumes that the incidence of HZ reaches a maximum in the last year of the study and the increase in HZ cases is solely an effect of the demographic change in the country

To estimate the expected number of cases up to year 2030 for both scenarios, the corresponding predicted value of incidence for each year was multiplied by the projected population estimate for same year. Population data and projections for the ≥65 year old age cohort up to 2030 were obtained from United Nations World Population Prospects: the 2015 revision [16]. The medium fertility variant population projection was used which takes into account a medium fertility, normal mortality and normal international migration rates.

Although annual incidence rates were presented for 11 studies [4–7, 9, 11, 17, 19–22], we focused on three studies as they reported the annual incidence data specifically for the ≥65 age group while also reporting incidence over a sufficiently long time period so as to perform linear best fit curve. The studies used to estimate the incidence rates were Leung et al. [6], Toyama et al. [7] and MacIntyre et al. [4] which reported HZ incidence rates for the US, Japan and Australia respectively and was adjudged to present a reasonable picture of the expected HZ burden in high-income country setting. For the US, both Leung et al. [6] and Hales et al. [11] evaluated the incidence rates for the ≥65 immunocompetent and overall population, but for our analysis we conservatively considered the former (note the incidence was consistently lower than rates reported in Hales et al.) as the yearly incidence data was readily available from the study Leung et al. [6] estimated the HZ incidence using a retrospective cohort study of medical claims data from the MarketScan (Truven Health Analytics) databases for 1993–2006. The MarketScan database is convenience sample that includes patient-level information from over 100 self-insured employers, state governments, hospitals, health insurance plans and Medicare from all states in the United States of America. HZ incidence (stratified by age and sex) was calculated using all persons with a first outpatient service associated with a 053.xx code (HZ International Classification of Diseases: ICD-9 code) as the numerator, and total MarketScan enrollment as the denominator. We did not include incidence data reported by Leung et al. [6] from 1993–1996 in our analysis as the MarketScan data during this period did not include Medicare data which represents the bulk of the US population aged 65 and above.

Toyama et al. [7] estimated the HZ incidence from the number of subjects that presented a new case of HZ who subsequently visited one of the 39 dermatology clinics and the dermatology departments of seven general hospitals belonging to the Miyazaki Dermatologic Society over a period of 10 years (1997–2006). Toyama et al. state that patients with HZ in Japan primarily consult dermatology clinics rather than practitioners of family medicine and, thus, the surveillance system covered nearly all the patients with HZ in the prefecture. HZ incidence was presented for the overall population segregated by age and gender.

MacIntyre et al. [4] presented the age specific HZ incidence based on the number of prescriptions of antivirals specific for the treatment of HZ. HZ incidence in the overall population was estimated from the number of antiviral prescriptions for HZ by adjusting the age specific numbers of prescriptions with the estimated proportion of new zoster cases that were prescribed direct-acting antivirals during the corresponding periods, derived from analyses of the Bettering the Evaluation and Care of Health (BEACH) database. The BEACH database is cross-sectional paper-based data collection of a nationally representative sample of general practitioners in Australia [4].

Yawn et al. estimated the HZ-attributable healthcare costs (in 2006 US dollars) for all patients with incident cases of HZ among residents of Olmsted County, Minnesota in the US [23]. To estimate the projected healthcare economic burden arising from HZ in those aged 65 and above for the US in 2030, we used the corresponding population estimates for the US and the projected HZ incidence obtained using scenario 1 (mentioned earlier). The 2006 US dollar healthcare costs were adjusted for the mid-year 2030 values by extrapolating the monthly US consumer price index for medical care trends from 2005–2015 using a linear best-fit [24]. It is assumed that the proportion of incident HZ cases that go on to develop sequelae (PHN and other non-pain complications) [3] remain constant over the time period of analysis.

All analyses were performed using *Microsoft Excel*.

## Results

### HZ Incidence

**Fig. 1:**
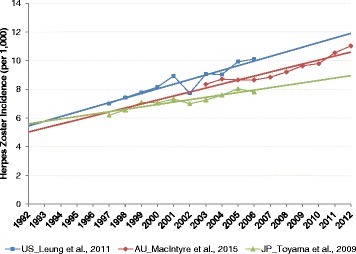
HZ incidence and trends in the Australia (AU), Japan (JP) and the United States (US)

The incidence data from the three countries showed an average annual increase of between 2.35% [7] (Japan; Toyama et al.) and 3.74% [6] (US; Leung et al.) over the corresponding study time periods (Fig. 1).

The incidence data for the three selected studies are shown below in Table 1.

**Table 1:** HZ incidence (per 1,000 persons) in the Australia, Japan and the United States

	1997	1998	1999	2000	2001	2002	2003	2004	2005	2006	2007	2008	2009	2010	2011	2012
Leung, 2011 (United States) [6]	7.01	7.42	7.80	8.16	8.94	7.73	9.06	9.04	9.93	10.11						
MacIntyre, 2015 (Australia) [4]							8.36	8.72	8.66	8.66	8.85	9.21	9.64	9.80	10.55	11.04
Toyama, 2009 (Japan) [7]	6.19	6.57	7.10	7.06	7.33	7.00	7.25	7.61	8.05	7.81						

### Demographics

The population projections for the ≥65 age cohort obtained from the United Nations data source are shown in Table 2. The elderly population for the US, Japan and Australia are expected to increase by 55, 10 and 53% respectively by the year 2030 compared to the levels in 2015. In 2001, 12.3% of the entire US population were aged ≥65; in 2030, that proportion is estimated to rise to 20.7%; a 68% increase over a period of 30 years. A similar trend is observed for Australia (Table 2). Japan, however, shows a much higher proportion of older adults (17.7% aged 65 and above in 2001) and the proportion is estimated to increase by 71.8% in 2030 to reach 30.4% of the total population. The proportion of the population aged ≥80 is expected to increase by 223 to 12.7% of the entire Japanese population in 2030.

**Table 2:** Population estimates for Australia, Japan and the United States

		United States		Japan		Australia	
	Age group	Population	% of total population	Population	% of total population	Population	% of total population
2001	All	285,796,198		125,974,298		19,308,681	
	>65	35,142,215	12.3	22,301,302	17.7	2,402,399	12.4
	>80	9,411,233	3.3	4,929,411	3.9	567,071	2.9
2015	All	321,773,631		126,573,481		23,968,973	
	>65	47,577,672	14.8	33,342,003	26.3	3,606,102	15.0
	>80	12,100,608	3.8	9,821,543	7.8	930,769	3.9
2030	All	355,764,967		120,127,264		28,481,570	
	>65	73,558,551	20.7	36,551,621	30.4	5,524,027	19.4
	>80	19,378,487	5.4	15,196,468	12.7	1,618,736	5.7

### Estimated incident HZ cases

In the first scenario, the number of incident HZ cases in the population aged ≥65 in the US is expected to increase by +343% (from 293,785 in 2001 to 1,303,328 in 2030) while the related increases in Japan and Australia are +176% (158,616 to 437,867) and +376% (18,105 to 86,268) respectively (Table 3).

**Table 3:** Projected annual incident cases of HZ in Australia, Japan and the United States in 2030

		United States			Japan			Australia		
		2001	2015	2030	2001	2015	2030	2001	2015	2030
Scenario 1	Projected Incidence (per 1,000)	8.36	12.88	17.72	7.11	9.46	11.98	7.54	11.44	15.62
	HZ cases in ≥65 years old	293,785	612,692	1,303,328	158,616	315,482	437,867	18,105	41,243	86,268
Scenario 2	Projected Incidence (per 1,000)	8.36	9.97	9.97	7.11	7.95	7.95	7.54	10.60	10.60
	HZ cases ≥65 years old	293,785	474,511	733,629	158,616	265,120	290,641	18,105	38,229	58,561

Scenario 1: linear extrapolation of HZ incidence trends observed during study period. Scenario 2: Last observation of incidence observed in the study period carried forward) HZ: Herpes Zoster

**Fig. 2:**
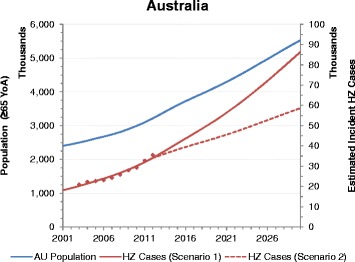
Population and HZ incident cases estimates up to 2030 for Australia (YoA: Years of Age, HZ: Herpes Zoster; AU: Australia)

**Fig. 3:**
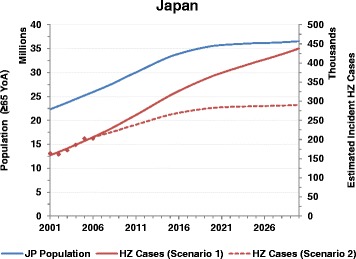
Population and HZ incident cases estimates up to 2030 for Japan (YoA: Years of Age, HZ: Herpes Zoster; JP: Japan)

**Fig. 4:**
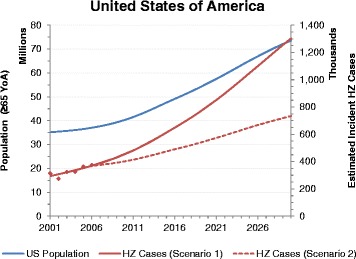
Population and HZ incident cases estimates up to 2030 for the United States (YoA: Years of Age, HZ: Herpes Zoster; JP: Japan)

In second scenario, the percentage increases from 2001 to 2030 for the US, Japan and Australia are +150% (293,785 to 733,629), +83% (158,616 to 290,641) and +223% (18,105 to 58,561) respectively (Table 3 and Figs. 2, 3 and 4).

### Estimated healthcare burden

HZ represents a significant burden each year to the healthcare system [23]. Yawn et al. estimated the annual HZ-related costs for US population to be around 1.1 Billion (B) US$. By adjusting the 2006 US$ values presented in the study to the projected 2030 US$ values (by extrapolating the published medical care cost index from January 2005 to December 2014 up to June 2030) [24], and combining it with the projected US population and HZ incidence for 2030, we estimated annual cost of HZ-related cases (assuming no vaccination) in the ≥65 age cohort to be approximately 4.74 Billion (B) US$ (Table 4).

**Table 4:** Estimated HZ-related healthcare cost burden for the ≥65 in the United States in 2030

	2005			2030		
	Cost/Case	Cases ('000 s)	Total HZ Costs (US$)	Cost/Case	Cases ('000 s)	Total HZ Costs (US$)
All HZ	$1,851	339	$627,773,213	$3,640	1,303	$4,743,559,326
HZ with PHN or Complications	$4,869	80	$389,153,306	$9,601	306	$2,937,397,732

*HZ* Herpes Zoster, *PHN* Post-herpetic Neuralgia, *US\* United States dollar

The complications of HZ (including PHN and other non-pain complications), which manifest in nearly 20–25% of HZ cases, cost nearly three times as much as an uncomplicated case of HZ [23]. Of the estimated 4.74B US$ HZ-associated costs in the US in 2030 in the ≥65 years old, 62% of the costs (2.94B US$) arise from the treatment of PHN and other complications (Table 4). Since the risk of developing HZ-associated complications (PHN and other non-pain complications) increases with age [3], the trend of the increasing proportion of the ≥80 population, especially in countries like Japan, is expected to further increase the economic burden of HZ.

## Discussion

Considering the simultaneous projected increase in the elderly population and the increase in HZ incidence, countries like the US, Japan and Australia are expected to see an increase of 110 and 213% in HZ cases between 2015 and 2030 in the ≥65 years old. The increase in both HZ incidence and the aging population is expected to be a global trend and thus the economic burden of HZ on healthcare systems is expected to be a global phenomenon as well.

There are limitations to the methodological approach undertaken in this study. This study used two scenarios to model and to project the temporal trend of HZ incidence in three chosen countries: a linear best-fit extrapolation of the reported annual incidence and a last observation carried forward method. A more rigorous approach would be to employ a time-series analysis and present the 95% confidence interval estimates for the predicted incidence in 2030. A Holt-Winters double exponential smoothing forecast of all three data-sets gave an estimate of HZ incidence similar to the incidence estimates obtained using the linear best-fit method we used. By using the two above-mentioned scenarios we feel that we present the range of possible outcomes of HZ incidence in the corresponding countries. Another limitation is that we used only one set of data for each country to predict HZ burden. Thus, the estimates may be potentially biased based on the study design and assumptions used in the corresponding studies. An alternative to this approach would have been to use meta-regression on the data obtained from all the studies. However, this would require a greater level of data (e.g. up to patent-level data in some cases) to factor out the variates present in the studies. Such an exercise would have to be undertaken to obtain a more accurate prediction of incidence. A better understanding of the underlying reasons for the increasing HZ incidence will also have to be studied to model trends. Various hypotheses have been postulated, but no consensus has been reached.

One of the reasons cited for this increase is the introduction of the varicella vaccination; [13] repeated exposure of the population to naturally circulating is thought to limit the occurrence of HZ through exogenous boosting of immunity to the varicella virus. However, both Hales et al. [11] and Russell et al. [5] have shown that the increasing trend of HZ incidence was evident even before the introduction of varicella vaccination in the US and Canada, respectively. The increased HZ incidence is also evident in countries without varicella vaccination programs or before the introduction of the vaccine [4, 7]. An assessment of impact modelling studies from various countries predicted that mass varicella vaccinations are expected to increase HZ incidence for approximately 50 years post-introduction of the vaccine [25]. However, real world evidence and modelling of the impact of varicella vaccination on HZ incidence is conflicting and further research is necessary to obtain a robust assessment.

In addition to the introduction of the public varicella vaccination programs, various other hypothetical factors have been attributed to the increase in HZ incidence such as, the aging population and associated chronic conditions and the increased prevalence of immunosuppressive conditions and treatments. Several studies, from various countries, suggest that age-specific HZ incidence has been increasing, i.e. the increasing incidence is observed across all age-groups [5, 11]. Also, a case-control study performed in the US [26] found that while the risk of HZ does increase with increasing number of chronic conditions, they do not ‘substantially explain’ why certain people go on to develop HZ.

Immunosenescence brought on by aging has been demonstrated to be associated with the reactivation of the latent VZV virus and thus the development of HZ [14]. However, Toyama et al. [7] found that the increase in HZ incidence was higher than the increase in the percentage of the older population. Increases in HZ incidence have also been observed across all age groups. It has been demonstrated that the risk of HZ is higher in subjects with immunosuppressive conditions or treatments. However, Leung et al. [6] found that the increase in HZ incidence was present in the ‘healthy’ population which excluded subjects that had any of the 200 identified immunosuppressive conditions or treatments.

The increasing trend of HZ incidence may also be due to changes in other risk factors previously identified for HZ, e.g. increasing physical limitation or psychological stress [14, 27]. Increases could also be attributed to better diagnosis through improved access to healthcare and public awareness. This increasing trend along with the concurrent aging of the population is expected to further strain healthcare budgets. The aging population coupled with low birth rates in many countries will also result in a decline in the proportion of the working-age population [16]. The ratios of the working working-age population (15–64 years old) to the older age group (65 and above) in the US in 2001 was 5.4 and is estimated to fall to 3.9 in 2020. A similar trend is observed for Australia and the corresponding ratios in Japan are 3.8 in 2001 to 2.1 in 2020.

A live attenuated Oka VZV vaccine which is licensed in various countries was shown to reduce HZ incidence by 51.1% (95% confidence interval, 44.2–57.6) [28]. However, vaccine efficacy was shown to reduce with age with the efficacy against HZ incidence dropping to 37.6% in those aged ≥70 [28]. Recent results of a phase III trial of an adjuvanted HZ subunit vaccine have demonstrated a substantial improvement in vaccine efficacy in the elderly with an efficacy of 97.2 (95% confidence interval, 93.7–99.0) in subjects ≥50 years of age [29] and 91.3% (95% confidence interval, 86.8–94.5) in subjects ≥70 years of age [30]. The two vaccines could help reduce the future global burden of HZ on the elderly.

## Conclusion

Due to the aging of the global population, a dramatic increase in the number of people suffering from HZ is expected over the next few decades unless more effective preventative action is taken.

## Abbreviations

HZ: Herpes Zoster
VZV: Varicella Zoster Virus
PHN: Post-herpetic Neuralgia
MeSH: Medical Subject Headings
BEACH: Bettering the Evaluation and Care of Health
US: United States
US$: United States dollar
